# Conditional survival and changing risk profile in patients with chordoma: a population-based longitudinal cohort study

**DOI:** 10.1186/s13018-019-1225-7

**Published:** 2019-06-17

**Authors:** Jin-Feng Huang, Dong Chen, Xuan-Qi Zheng, Jia-Liang Lin, Xiang-Yang Wang, Ai-Min Wu

**Affiliations:** Department of Spine Surgery, Zhejiang Spine Surgery Centre, Orthopaedic Hospital, The Second Affiliated Hospital and Yuying Children’s Hospital of Wenzhou Medical University, 109# Xueyuan Road, Wenzhou, Zhejiang, 325027 China

**Keywords:** Chordoma, Conditional survival, Survival, Changing risk profile

## Abstract

**Objective:**

To evaluate the conditional survival of patients with chordoma to potentially help physician planning of optimal cancer surveillance and guide better clinical decisions.

**Methods:**

In total, 1942 patients with chordoma were identified and extracted from Surveillance, Epidemiology, and End Results (SEER) databases (1973–2015). The cumulative survival estimates were used to calculate the conditional survival rate, and the Greenwood formula was used to estimate the 95% CI. In addition, multivariable Cox regression analyses were used to calculate hazard ratios, according to the duration of survival.

**Results:**

The conditional 5-year disease-specific survival in patients with regional or localized chordoma was relatively stable over time, whereas in patients with distant chordoma, there was a gradual improvement. The conditional 5-year disease-specific survival (DSS) of patients older than 60 years old and patients with a tumor size between 5 and 10 cm improved. Interestingly, for patients with a tumor larger than 10 cm, the conditional 5-year DSS decreased over time. After surviving 5 years, the hazard ratio (HR) of patients older than 60 years old decreased from 1.33 to 1.24, that of patients with a tumor size between 5 and 10 cm decreased from 1.61 to 1.52 and that of patients with distant metastasis decreased from 3.30 to 1.09. However, after surviving 5 years, the HR of patients with a tumor size larger than 10 cm increased from 2.33 to 3.77, that of patients who underwent surgical resection increased from 0.37 to 0.58 and that of patients who received radiation therapy increased from 0.81 to 1.04.

**Conclusion:**

Age at diagnosis, tumor size and disease stage can influence conditional survival for patients with chordoma. The HR of different factors will change over the survival time. Therefore, understanding the changing risk profile and conditional 5-year DSS of chordoma is critical for accurate clinical treatment guidance.

## Introduction

Chordoma is a kind of bone tumor, which is rare, relatively slow-growing, and a low-grade malignancy, but it is locally invasive and aggressive [1, 2]. Chordomas arise from the remnants of the notochord and usually occur within the axial skeleton [3]. In approximately 50 to 60% of cases, the primary site is the sacrum. In 30% of cases, the tumor location is in the skull base region, and in 15% of cases, the tumor is located in the vertebrae [2, 4, 5].

The prevalence of chordoma is less than 1 per 1,000,000 people [6]. The incidence of chordoma peaks at 50–60 years of age and is very unlikely in patients younger than 40 years old [1, 7–9]. In addition, it is more common in men [10]. The recurrence rate is very high, especially for tumors located above the sacral 3 level [11]. Most chordomas are located proximal to neurovascular structures, and the recurrence rate is very high, which may lead to poor prognoses [12].

The margin of resection is closely related to local recurrence [13]. Data from several studies suggest that a wide margin resection can reduce the recurrence rate and prolong survival time [14, 15]. Therefore, the primary treatment for this tumor is total or near-total resection with or without radiation therapy [16–18]. However, total or near-total resection is difficult, since these tumors impinge on vascular and neural structures [19]. Recently, Hua used radiofrequency ablation, which can ensure tumor-free exposed margins, combined with gross total excision in the resection of a C4 cervical chordoma, which can improve the local relapse-free survival [20]. It might be a promising method. The survival rate is usually used to estimate cancer mortality. Though the survival rate is effective for assessing a patient’s prognosis, it provides little information about the changing risk profile as time passes [21]. Patients diagnosed with distant metastases, who usually die quickly, severely affect the survival curve [22]. Conditional survival can play an important role in addressing this issue [23]. Conditional survival, which may be more accurate to assess future patients’ outcomes and prognoses, means the survival in patients who have already survived a period of time [24–27].

Previous surveys have shown that conditional survival can help physicians plan optimal cancer surveillance and guide better clinical decisions [22, 28]. Therefore, the conditional survival has substantial value for patients and medical professionals [29]. However, previous studies have only provided the survival rate of patients with chordoma [6, 30]. To our knowledge, no previous study has attempted to define the conditional survival of chordoma. Therefore, the aim of this study was to evaluate the conditional survival of patients with chordoma to potentially help physician planning of optimal cancer surveillance and to guide better clinical decisions.

## Patients and methods

The Surveillance, Epidemiology, and End Results (SEER) Program, covering approximately 28% of the US population across 18 different regions, is supported by the National Cancer Institute of the USA and has provided statistical information on tumor cases since 1973. The SEER registry is a validated database that is frequently applied in cancer survival studies. In this study, we used the SEER registry to extract data for patients who were diagnosed with chordoma from 1973 to 2015 to calculate the conditional 5-year disease-specific survival (DSS).

Patients in the SEER registry diagnosed with chordoma from 1973 to 2015 were identified using the Histologic International Classification of Disease for Oncology, 3rd edition (IDO-O-3) codes 9370 to 9372, which included chordoma not otherwise specified (NOS), chondroid chordoma, and dedifferentiated chordoma. Information, including sex, age, race, year of diagnosis, primary tumor site, tumor size, disease stage, treatment method, marital status, and follow-up information, was recoded. Tumor stage was categorized according to SEER summary stage as localized, regional, or distant. We assigned patients to two groups: less than 60 years old and over 60 years old. The primary endpoint was DSS, defined as the time from diagnosis to death from chordoma, as determined from SEER data. The raw data in this study was downloaded from the SEER web site (https://seer.cancer.gov/data/) via SEER*Stat in client-server mode after we submitted a request for access and signed the SEER research data agreement.

Conditional survival was defined as the probability of DSS at 5 years from the day of diagnosis, given that the patient had already survived for a period of time (× years) [24].

$$ \mathrm{disease}-\mathrm{specific}\ \mathrm{survival}\ \left(y|x\right)=\frac{\mathrm{DSS}\left(x+y\right)}{DSS(x)} $$

For instance, to calculate the 5-year DSS rate for a patient who has already survived 1 year (*x* = 1, *y* = 5), the 5-year conditional DSS rate, DSS (1 + 5), is divided by the 1-year DSS rate, DSS (1).

### Statistical analysis

**Fig. 1:**
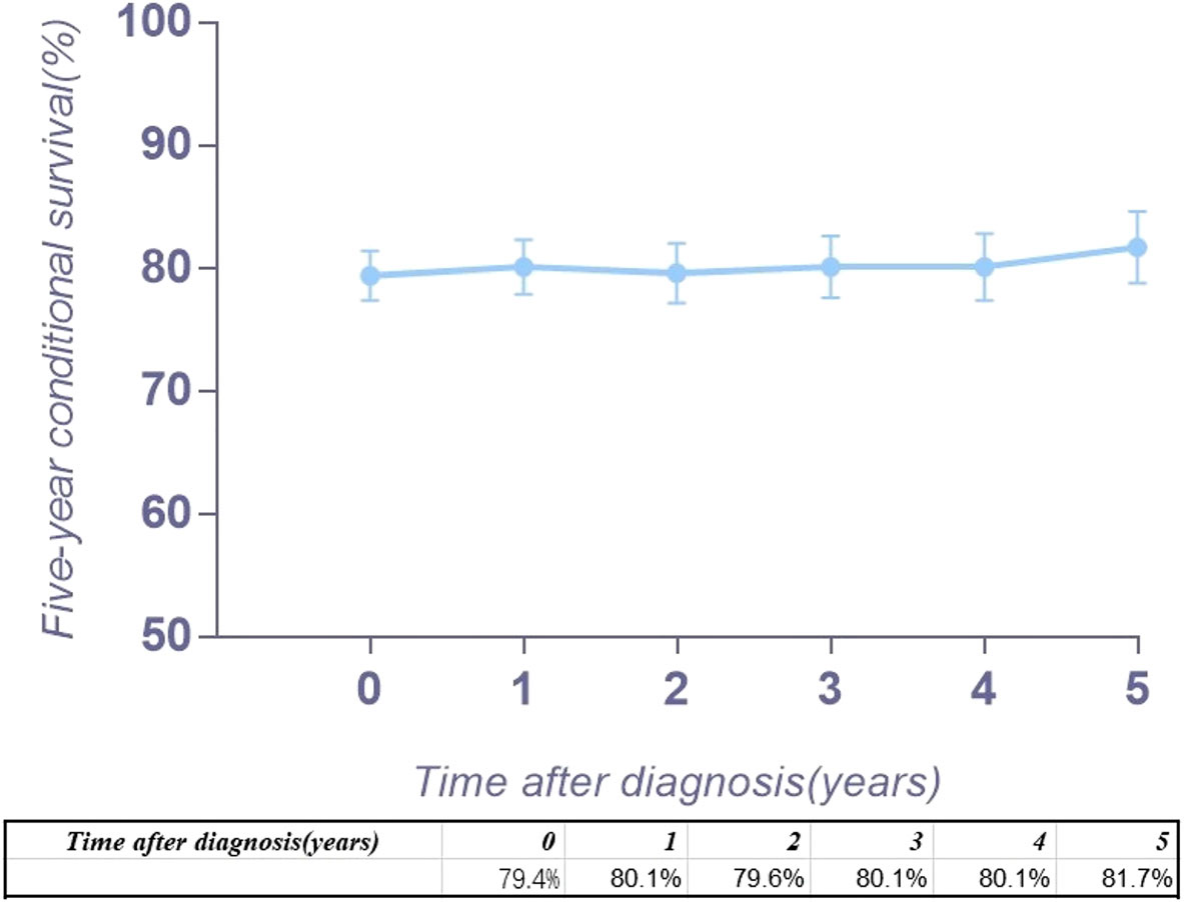
Kaplan–Meier survival curves illustrating the chordoma conditional 5-year disease-specific survival

We extracted data for patients with chordoma from the SEER database. The year of diagnosis was converted into a categorical variable of 1980s, 1990s, 2000s, and 2010s. Then, we calculated the 5-year survival rates for all patients at risk at the time of diagnosis and contingent on 1, 2, 3, 4, and 5 years of survival. The Kaplan–Meier method was used to calculate the conditional survival rate, and the Greenwood formula was used to estimate the 95% confidence interval (CI) and then to draw the survival curves (Fig. 1). The changing effect of the risk was examined with multivariable Cox regression analyses for the entire population, in patients who survived 1, 2, 3, 4, and 5 years, and the 95% CI.

All statistical analyses were performed with the SPSS software (version 18; IBM Corp., USA) and STATA software (version 14.2; Stata Corp, College Station, TX).

## Results

Data for a total of 1942 patients from 1973 to 2015 with chordoma were collected and analyzed. Table 1 shows the distribution of the patients’ characteristics in the study. The median age (range) of diagnosis was 57 (0–98) years, and the mean age at diagnosis was 55.0 ± 19.9 years, with a higher incidence in male patients (58.7%). The majority (86.3%) of tumors were found in white patients, while 3.6% of tumors were found in black patients. Regarding tumor size, 28.7% of patients had a tumor less than 5 cm; 19.2%, more than 5 cm and less than 10 cm; 9.0%, greater than 10 cm; and 43.1%, unknown. The majority of patients did not have distant metastasis (68.2%), while 7.3% of patients had distant metastasis and 24.5% of patients had an unknown status of distant metastasis. For treatment, 78.4% underwent surgery, and 44.1% received radiation therapy. Using Kaplan–Meier analysis, disease-specific survival (DSS) was 86.8% at 3 years, 79.3% at 5 years, and 64.8% at 10 years.

**Table 1:** Patient cohort characteristics

Characteristic	Value
Age, years	
Mean ± SD	55.0 ± 19.9
Median (range)	57(0–98)
Interquartile range	28
Age, *n* (%)	
0–59	1067(54.9%)
60+	875(45.1%)
Year of diagnosis, *n* (%)	
1970s	93(4.8%)
1980s	188(9.7%)
1990s	308(15.9%)
2000s	752(38.7%)
2010s	604(31.1%)
Gender, *n* (%)	
Male	1139(58.7%)
Female	803(41.3%)
Race, *n* (%)	
White	1675(86.3%)
Black	69(3.6%)
Other	180(9.3%)
Unknown	18(0.9%)
Histological type, *n* (%)	
Chordoma, NOS	1840(94.7%)
Chondroid chordoma	90(4.6%)
Dedifferentiated chordoma	12(0.6%)
Disease stage, *n* (%)	
Localized	625(32.2%)
Regional	700(36.0%)
Distant	142(7.3%)
Unknown	475(24.5%)
Tumor size, *n* (%)	
< 5	557(28.7%)
5~10	373(19.2%)
≥ 10	175(9.0%)
Unknown	837(43.1%)
Surgery, *n* (%)	
Yes	1523(78.4%)
No	419(21.6%)
Radiation, *n* (%)	
Yes	856(44.1%)
No	1035(53.3%)
Unknown	51(2.6%)

*NOS* not otherwise specified

**Fig. 2:**
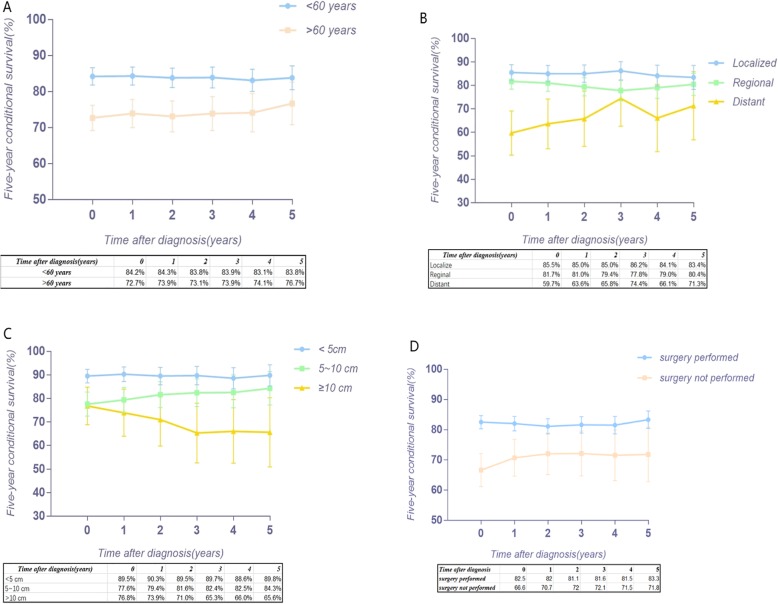
Conditional 5-year disease-specific survival of patients **a** who were less than 60 years old and over 60 years old with chordoma; **b** who had localized, regional, and distant chordoma; **c** who had different tumor sizes; and **d** who underwent or did not undergo surgical therapy

**Fig. 3:**
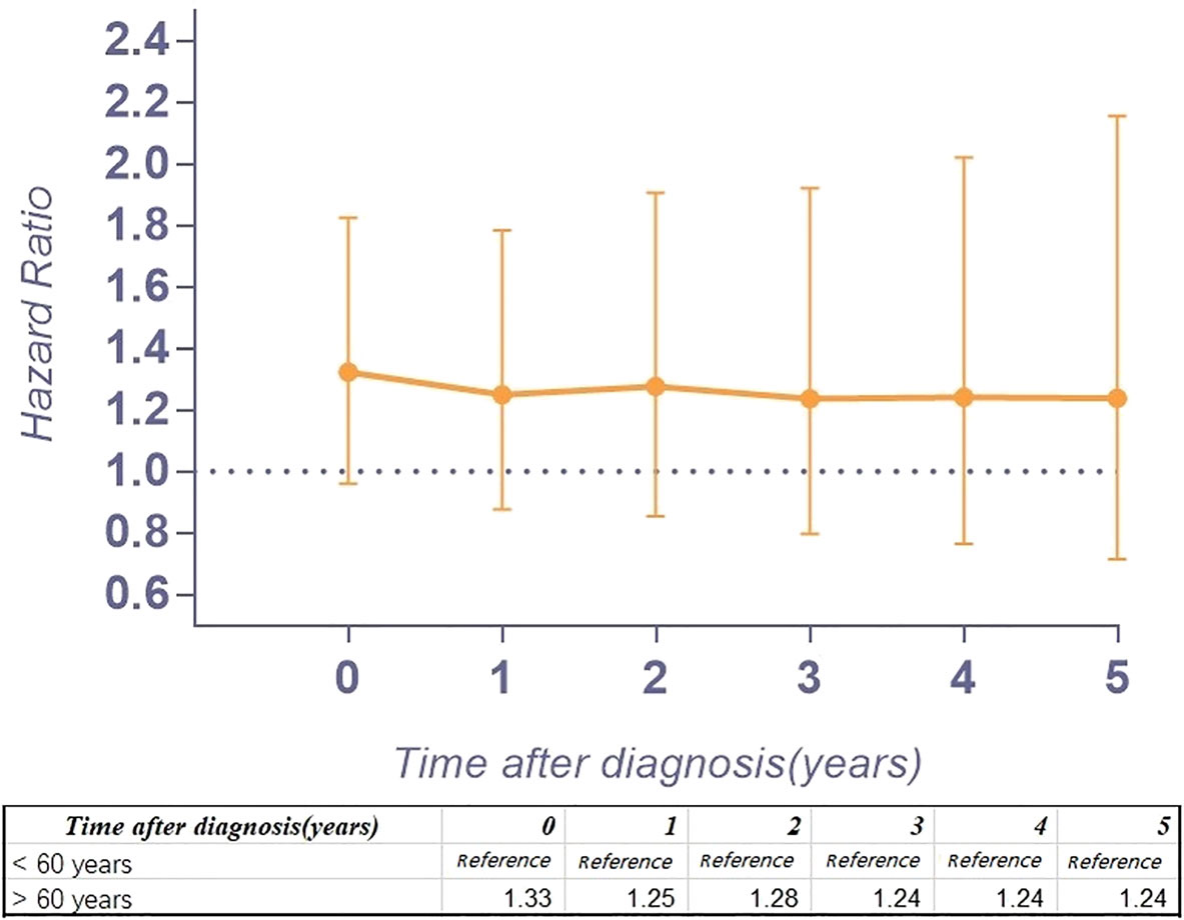
Hazard ratios from multivariable Cox regression analyses for prediction of disease-specific mortality with the number of years of survival stratified according to age (< 60 years old is the reference.)

**Fig. 4:**
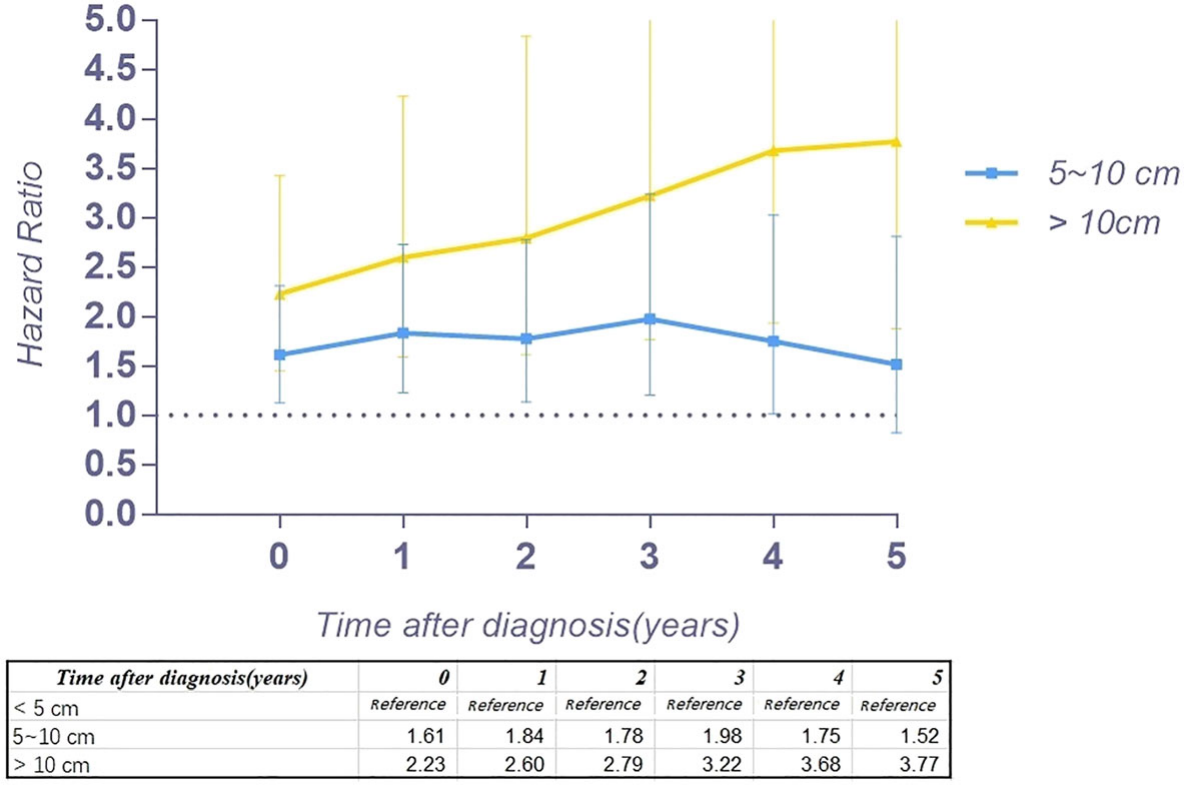
Hazard ratios from multivariable Cox regression analyses for prediction of disease-specific mortality with the number of years survived stratified according to tumor size (tumor size < 5 cm is the reference)

**Fig. 5:**
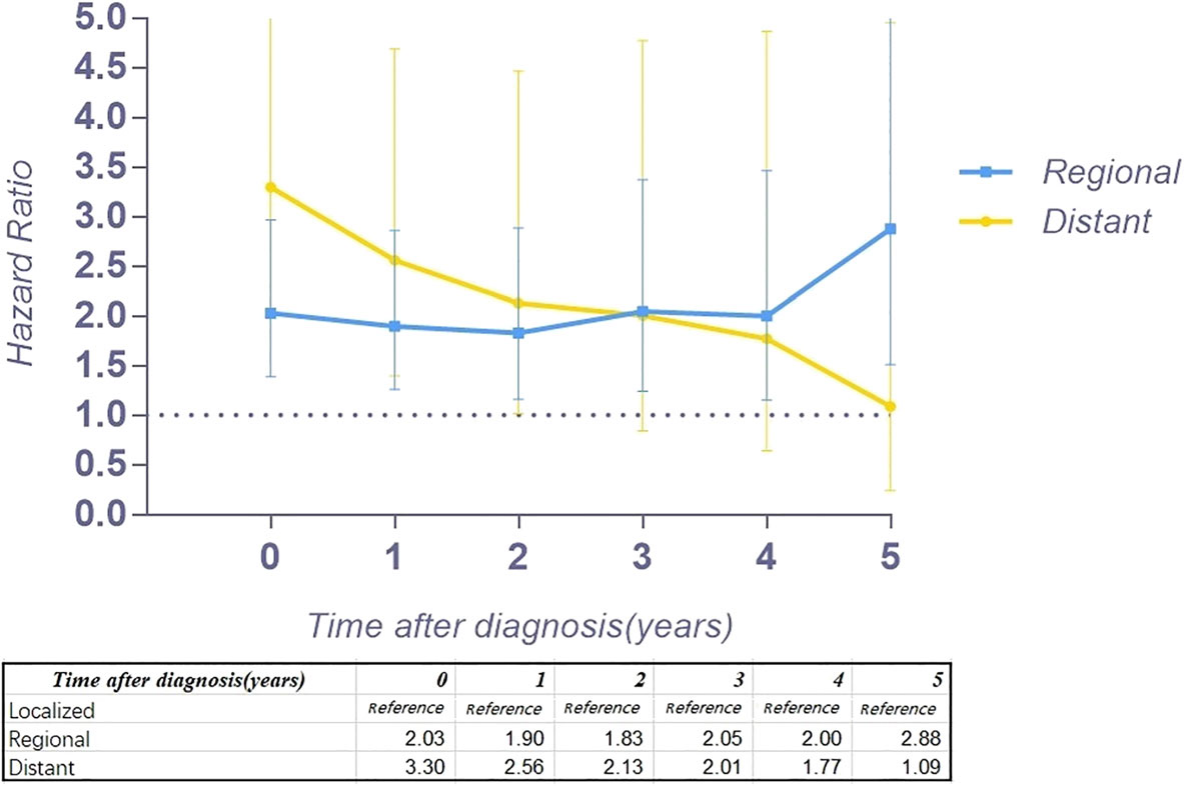
Hazard ratios from multivariable Cox regression analyses for prediction of disease-specific mortality with the number of years survived stratified according to disease stage (localized tumor is the reference)

**Fig. 6:**
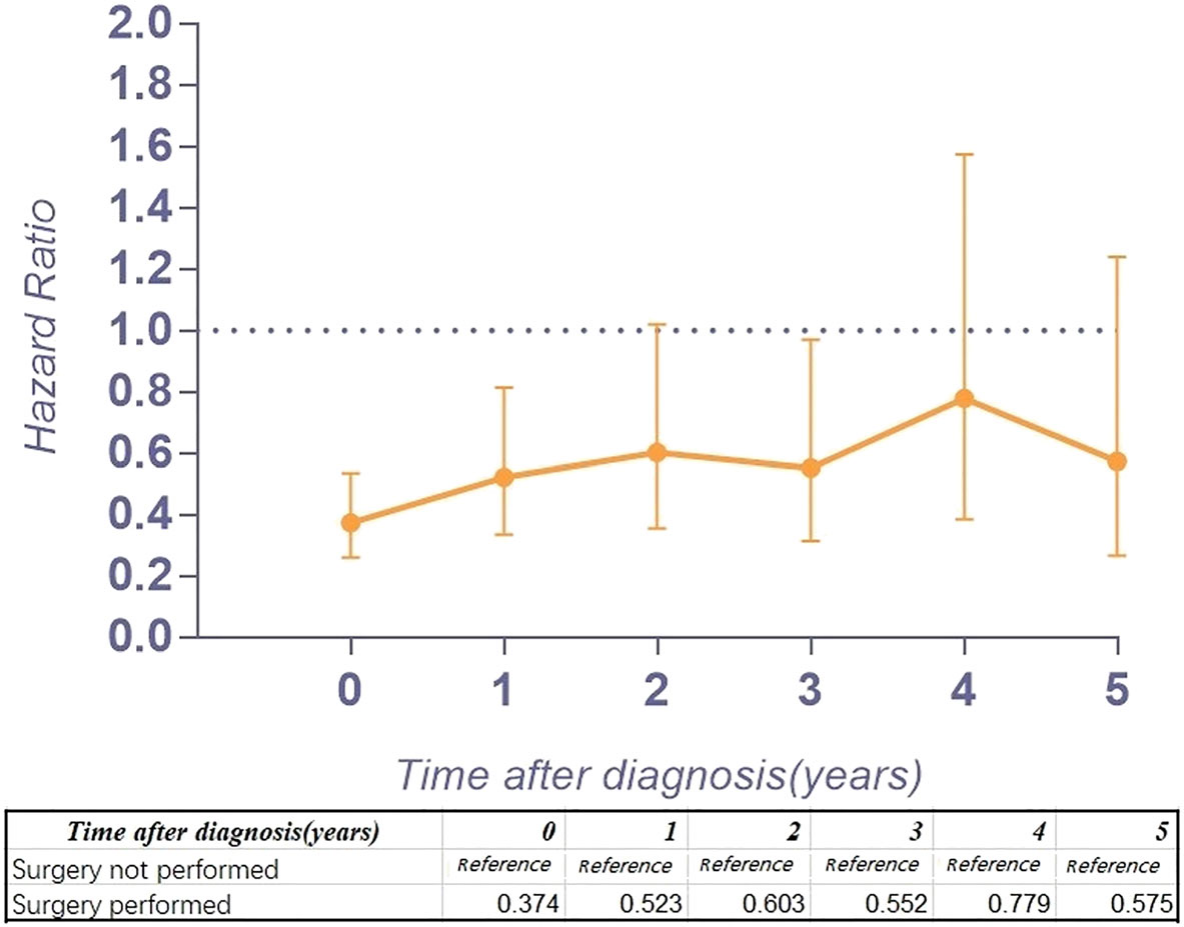
Hazard ratios from multivariable Cox regression analyses for prediction of disease-specific mortality with the number of years survived stratified according to surgical therapy (surgery not performed is the reference)

**Fig. 7:**
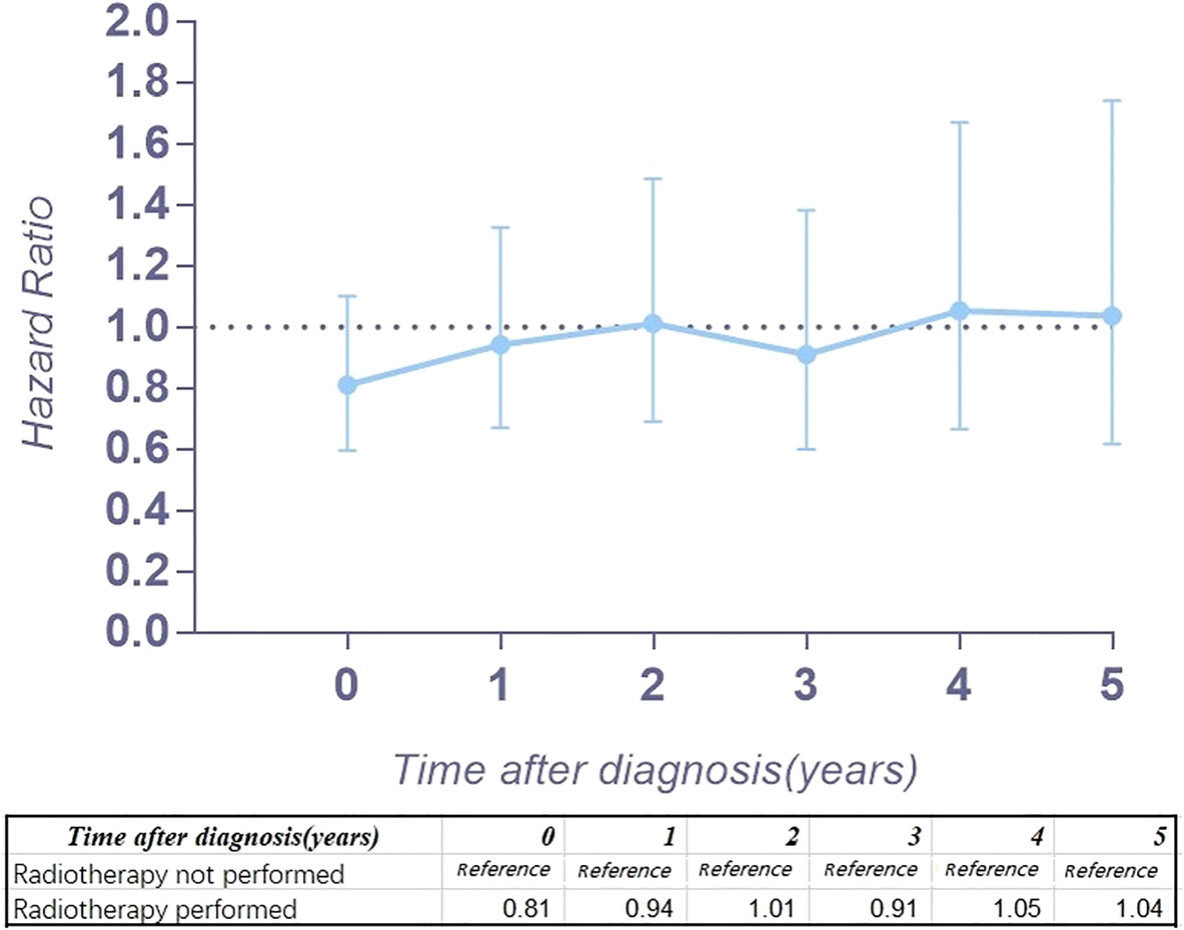
Hazard ratios from multivariable Cox regression analyses for prediction of disease-specific mortality with the number of years survived stratified according to radiation therapy (radiotherapy not performed is the reference)

Figure 2 a provides the conditional 5-year disease-specific survival based on the age of patients. Figure 2 b shows the conditional 5-year disease-specific survival of patients with localized, regional, or distant metastases. The conditional 5-year disease-specific survival of patients whose tumor size was less than 5 cm, between 5 and 10 cm, and larger than 10 cm is illustrated in Fig. 2c, and the conditional 5-year disease-specific survival of patients who underwent or did not undergo surgery is shown in Fig. 2d. The HR decreased in patients who were older than 60 years old (HR, from 1.33 to 1.24, Fig. 3), had a tumor between 5 cm and 10 cm (HR, from 1.61 to 1.52, Fig. 4), and had distant metastasis (HR, from 3.30 to 1.09, Fig. 5). However, the HR increased in patients with a tumor larger than 10 cm (HR, from 2.23 to 3.77, Fig. 4) and patients who underwent surgical resection (HR, from 0.37 to 0.58, Fig. 6) or radiation therapy (HR, from 0.81 to 1.04, Fig. 7).

## Discussion

This study aimed to provide conditional survival data for patients with chordoma who had already survived a specific period of time. Previous studies only provided the survival rate of patients with chordoma [6, 30]. Previous studies have demonstrated that the conditional survival rate can provide critical quantitative information and more reliable clinical guidance for patients and clinicians [29, 31, 32]. Therefore, this study has a very important role in predicting the survival rate of patients who have already survived for several years. In this study, for patients with regional or localized chordoma, the conditional 5-year DSS was surprisingly relatively stable over time, whereas for distant chordoma, there was a gradual improvement in the conditional 5-year DSS (+ 11.6%). Therefore, when patients with metastatic chordoma have survived five years, their 5-year DSS improved significantly, which could have a positive effect in these patients because it could help clinicians make more accurate decisions for further treatment. The 5-year DSS of patients older than 60 years old improved (+ 4%) after survival for 5 years. For patients who underwent surgery, the 5-year DSS was stable. However, for patients who did not undergo surgery, the 5-year DSS improved gradually. For patients with different tumor sizes, the results differ widely. For patients with a tumor less than 5 cm, the conditional 5-year DSS was steady, whereas for patients with a tumor between 5 and 10 cm, the conditional 5-year DSS improved as survival time gradually increased (+ 6.7%). One unanticipated finding was that for patients with a tumor larger than 10 cm, the conditional 5-year DSS decreased as time passed (− 11.2%), which has significant clinical value and provides guidance for clinicians. This result has not previously been described. A possible explanation for this result might be that the tumor is too large to perform complete surgical resection, which directly impacts the survival.

Therefore, the conditional 5-year DSS can generate a positive influence on patients having an overall poorer prognosis. The induction of the conditional 5-year DSS will encourage patients more and provide more accuracy for clinicians to estimate prognoses and explain the situations patients will face.

Because the HR at different times varies, our study assessed the risk profile of multiple variables at different timepoints to determine the change in the hazard ratio of multiple variables over time, which might provide more substantial guidance than traditional hazard ratio analysis. In our study, we found that the HR for age greater than 60 years old decreased slightly over time. Interestingly, the HR of tumor size between 5 and 10 cm increased 4 years after diagnosis, and after surviving 5 years, it decreased instead. However, the HR of tumor size larger than 10 cm increased gradually over time (from 2.23 to 3.77), which means the risk for tumor size gradually increases after diagnosis. In addition, the HR of patients with regional tumors first decreased and then increased. Surprisingly, the HR of distant metastasis decreased substantially (from 3.30 to 1.09) over time after diagnosis. For patients with metastatic tumors, the threat to the survival rate of this risk factor reduced after survival for several years. However, the timing of performing surgery or radiation therapy will affect the survival of patients. The HR increasing over time after surgery or radiation therapy provides important clinical guidance. The earlier surgery or radiation therapy is performed, the lower the risk for mortality. Therefore, for patients with chordoma, it is imperative to perform surgery or radiation therapy as early as possible.

Our study also has some limitations. First, our findings are derived from retrospective data, which is similar to all observational studies. This element may associate the results with unavoidable selection and attribution biases. Second, the SEER database does not provide surgical resection type, which should be included in future studies. Third, the effectiveness of novel radiation techniques could not be assessed.

## Conclusion

Age at diagnosis, tumor size, and disease stage can influence the conditional survival for patients with chordoma. The trend of conditional survival differs in patients with different states. The HRs of different factors change over the survival time. Therefore, understanding the changing risk profile and conditional 5-year disease-specific survival of chordoma is critical for accurate clinical treatment guidance.

## Data Availability

Not applicable.

## Abbreviations

CI: Confidence interval
DSS: Disease-specific survival
HR: Hazard ratio
IDO-O-3: International Classification of Disease for Oncology, 3rd Edition
NOS: Not otherwise specified
SEER: Surveillance, Epidemiology, and End Results
